# Supplementary material for: The causal relationship between the human gut microbiota and pyogenic arthritis: a Mendelian randomization study
Source: Front Cell Infect Microbiol. 2024 Nov 26;14:1452480. doi: 10.3389/fcimb.2024.1452480 (PMC11629706; doi:10.3389/fcimb.2024.1452480)
Supplement: Supplementary file 1 [file DataSheet1.zip › mendelian test/ebi-a-GCST90027666.csv_forest.pdf]

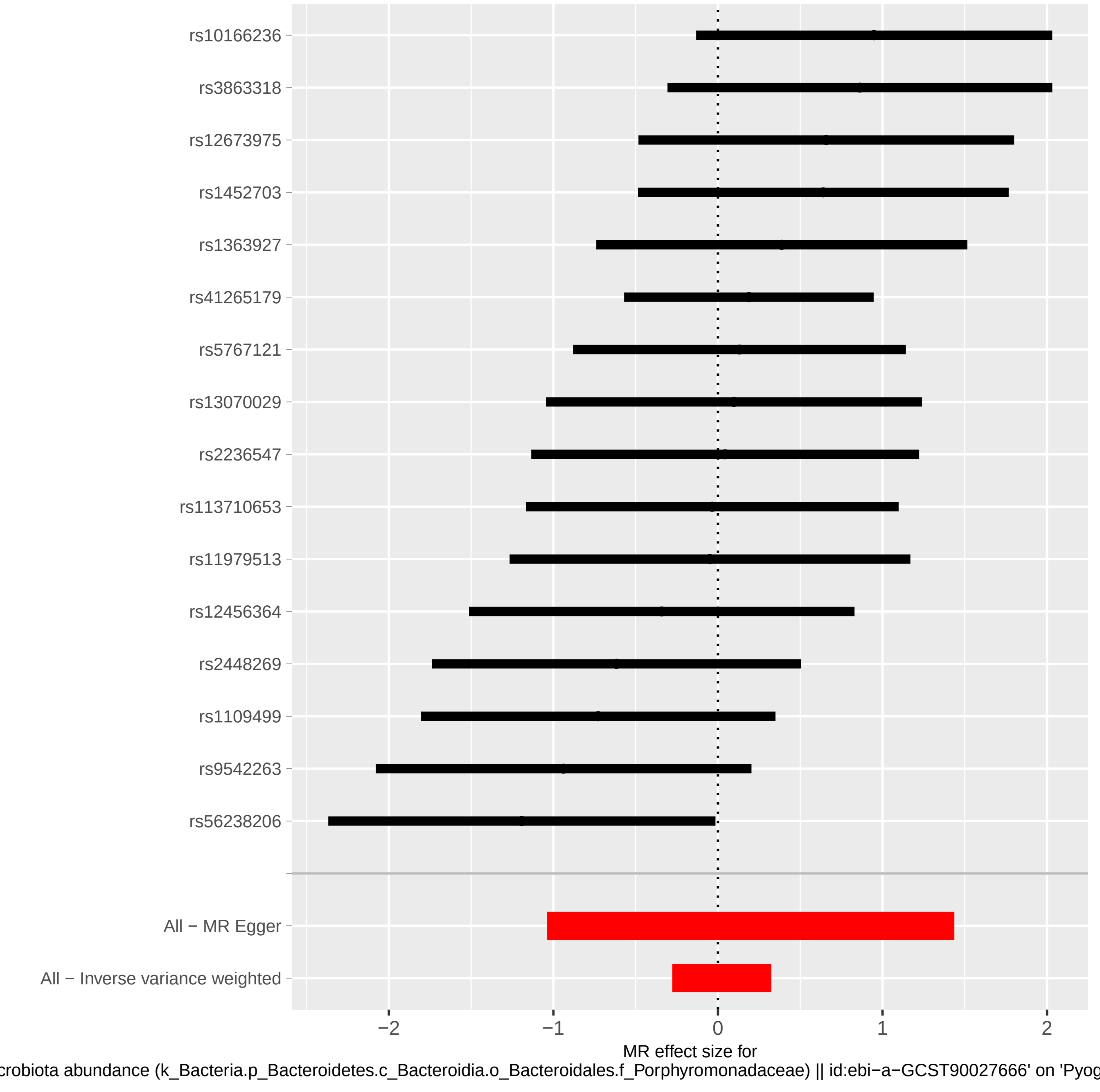

rs10166236
rs3863318
rs12673975
rs1452703
rs1363927
rs41265179
rs5767121
rs13070029
rs2236547
rs113710653
rs11979513
rs12456364
rs2448269
rs1109499
rs9542263
rs56238206
All − MR Egger
All − Inverse variance weighted
−2
−1
0
1
2
MR effect size for
crobiota abundance (k_Bacteria.p_Bacteroidetes.c_Bacteroidia.o_Bacteroidales.f_Porphyromonadaceae) || id:ebi−a−GCST90027666' on 'Pyog
